# Minimally invasive thymectomy: comparative analysis of short-term outcomes after video-assisted thoracoscopy and two robotic platforms

**DOI:** 10.3389/fsurg.2026.1836452

**Published:** 2026-05-25

**Authors:** Ilaria Righi, Davide Tosi, Riccardo Orlandi, Margherita Brivio, Francesco Damarco, Margherita Cattaneo, Paolo Mendogni, Sonia Di Tella, Mario Nosotti, Lorenzo Rosso

**Affiliations:** 1 Thoracic Surgery and Lung Transplantation Unit, Fondazione IRCCS Ca' Granda-Ospedale Maggiore Policlinico, Milan, Italy; 2 School of Thoracic Surgery, University of Milan, Milan, Italy; 3 Experimental and Applied Psychology Laboratory, Department of Health and Life Sciences, Università Europea di Roma, Rome, Italy; 4 Department of Pathophysiology and Transplantation, University of Milan, Milan, Italy

**Keywords:** Da Vinci®, outcomes, rats, thymectomy, VATS, versius

## Abstract

**Objective:**

Minimally invasive thymectomy has largely replaced the traditional trans-sternal approach. Among the minimally invasive strategies, video-assisted thoracoscopic and robot-assisted thoracoscopic procedures are the most frequently used. The introduction of new robotic platforms has renewed interest in comparing different systems. This study aimed to evaluate short-term outcomes, learning curves, and surgeon workload in thymectomy performed with a video-assisted thoracoscopic technique, the Da Vinci robotic system, and the Versius robotic system.

**Methods:**

A retrospective, comparative study was conducted on 90 consecutive patients who underwent minimally invasive thymectomy between 2016 and 2025 at a single tertiary center. Thirty procedures were performed by a video-assisted thoracoscopic approach (2016–2020), and sixty with robotic assistance (2021–2025), equally divided between Da Vinci and Versius systems. The primary endpoint was postoperative hospital stay. Secondary endpoints included operative time, postoperative analgesic use, complications, conversion rate, and chest tube duration. Surgeon workload was evaluated using a validated multidimensional questionnaire assessing mental, physical, and temporal demands, performance, effort, and frustration.

**Results:**

Hospital stay was significantly shorter after robotic thymectomy compared with the video-assisted approach (3.2 ± 1.0 days for Da Vinci and 3.0 ± 1.2 days for Versius vs. 4.5 ± 1.9 days; *p* < 0.001). Secondary outcomes were comparable among the three techniques. Cumulative sum analysis demonstrated faster procedural stabilization with robotic systems. Workload assessment indicated higher perceived demand with the Versius system, mainly in mental and physical domains.

**Conclusions:**

In this single-center experience, robotic thymectomy was associated with shorter hospital stay and earlier procedural stabilization compared with VATS, with similar perioperative safety. Differences between robotic platforms were mainly observed in surgeon-reported workload. These findings should be interpreted within the context of surgeon experience and institutional evolution.

## Introduction

Thymectomy has undergone a profound evolution over the past three decades. The traditional open trans-sternal approach, once considered the gold standard, has progressively been abandoned, except in cases of very large or invasive thymic tumors. Minimally invasive strategies have since become the preferred modalities, primarily due to their association with reduced surgical trauma, shorter hospitalization, and improved postoperative recovery (1).

Among these techniques, video-assisted thoracoscopic surgery (VATS) and robot-assisted thoracoscopic surgery (RATS) have gained increasing popularity. VATS thymectomy was the first minimally invasive alternative to open surgery and has been widely adopted. In 2015, Xie and colleagues (2) published a systematic review evaluating the efficacy and safety of VATS versus open thymectomy: VATS was associated with reduced intraoperative blood loss, shorter hospital stays, and lower rates of postoperative pneumonia. Nevertheless, VATS is known to present a relatively long and heterogeneous learning curve (3, 4).

Conversely, RATS thymectomy, initially introduced with the Da Vinci platform, rapidly established itself as a safe and effective procedure, providing enhanced dexterity, three-dimensional visualization, and improved ergonomics for the surgeon (5). Several studies have demonstrated a steep yet manageable learning curve for RATS, with reproducible outcomes even during the early adoption phase (6).

More recently, the introduction of alternative robotic platforms such as the Versius system has further expanded the landscape of minimally invasive thymic surgery (7). The entry of new technologies into the market has stimulated direct comparison between different robotic modalities and between robotic and VATS approaches. These comparisons may reveal differences in clinical outcomes, learning curves, and broader implications for the adoption of robotic surgery in routine practice.

The present work aims to provide a comparative analysis of thymectomy performed via VATS, RATS with the Da Vinci system, and RATS with the Versius platform. By addressing the strengths and limitations of each approach, as well as their learning curves and early perioperative outcomes, this study seeks to clarify the evolving role of robotic platforms in thymic surgery and to contribute to the discussion on the most appropriate minimally invasive strategy for thymectomy.

## Patients and methods

### Study design and population

This was a retrospective, comparative study including consecutive patients who underwent thymectomy between 2016 and 2025 at the Foundation IRCCS Ca' Granda Ospedale Maggiore Policlinico. Inclusion criteria were: preoperative imaging demonstrating an anterior mediastinal mass suspected to be a thymoma clinical stage I (T1a, 9th TNM edition) or thymic hyperplasia in patients with myasthenia gravis, age ≥18 years, American Society of Anesthesiologists (ASA) physical status classification ≤4, and provision of written informed consent. Exclusion criteria included: radiologic evidence of invasion into adjacent organs, previous thoracic surgery, or any surgical access other than the lateral transthoracic approach. During the first study period (2016–2020), selected cases were the first performed using a video-assisted thoracoscopic approach (VATS, *n* = 30). From 2021 onward, all minimally invasive thymectomies were performed exclusively with robotic assistance and were equally distributed between the Da Vinci Xi (*n* = 30) and Versius (*n* = 30) platforms.

### Surgical teams and perioperative management

Four consultant thoracic surgeons performed all the minimally invasive procedure included in this study: all these surgeons started together their experience with VATS thymectomy. From 2021 the same surgeons, starting from a proficiency level in VATS thymectomy, began together the robotic learning curve: each robotic platform was handled exclusively by two dedicated surgeons. Nursing staff, anesthesiologists, and operating room technicians were the same for all three approaches, ensuring uniform perioperative management. The surgical approach was left-sided in 10% of VATS, 6.7% of Da Vinci, and 6.7% of Versius cases. At our institution, the lateral transthoracic intercostal approach represented the standard minimally invasive access during the study period. This preference reflected institutional experience, team familiarity with the technique, and the need to maintain a consistent operative setup across VATS and both robotic platforms. Although the subxiphoid approach is an important and increasingly adopted alternative, it was not part of routine practice during the study period.

Postoperative care pathways, including analgesic protocols, chest tube management, mobilization, and discharge criteria, remained unchanged throughout the study period. Conversion to open surgery was defined as the need for thoracotomy in all groups. Analgesic regimens were converted to morphine equivalent (ME) doses to allow consistent comparison. Surgical techniques are detailed in the Supplementary Materials. Briefly, all the procedures were performed through three-port unilateral transthoracic approach with carbon dioxide insufflation.

### Endpoints

The primary endpoint was postoperative length of stay (LOS). Secondary endpoints included operative time (skin incision to closure), postoperative analgesic consumption (reported in ME per day), postoperative complications [graded according to the modified Clavien–Dindo classification (8)], chest tube duration, and the rate of conversion to open or alternative minimally invasive surgery.

### Data collection

Clinical, operative, and postoperative data were prospectively collected in an institutional database and retrospectively analyzed for the present study. Demographic characteristics included age, sex, smoking status, ASA score, Charlson comorbidity index, diagnosis, and thymoma stage.

### Surgeon workload assessment

In addition to clinical and perioperative outcomes, surgeon workload was evaluated using the NASA Task Load Index (NASA-TLX) questionnaire (Supplementary Figure S1) (9). All the involved consultant surgeons (two operating with the Da Vinci system and two with the Versius platform) completed the questionnaire following their initial robotic thymectomy cases, corresponding to the cumulative sum (CUSUM) learning phase. Responses were used to quantify perceived workload for each robotic system and to explore potential associations with learning-curve dynamics. Given that each robotic platform was used by a dedicated pair of surgeons, surgeon-related factors may have influenced both learning-curve dynamics and workload perception.

### Statistical analysis

Continuous variables were expressed as mean ± standard deviation (SD) and compared using one-way ANOVA or the Kruskal–Wallis test, as appropriate. Categorical variables were presented as frequencies and percentages and compared using the chi-square or Fisher's exact test. A sample size calculation was performed based on the primary endpoint – LOS – using G*Power (version 3.1). Assuming a mean difference of 1 day between groups as clinically relevant, a common standard deviation of 1.2 days (based on institutional historical data), an *α* error of 0.05, and a statistical power of 80%, a minimum of 27 patients per group was required. To account for potential dropouts or missing data, 30 patients were included in each group. A propensity score–based inverse probability of treatment weighting (IPTW) approach was applied to adjust for potential baseline imbalances among groups. The propensity score was estimated using a multinominal logistic regression model including the following covariates: age, sex, comorbidities, ASA score, presence of myasthenia gravis, and surgical team. Standardized mean differences were used to assess covariate balance after weighting. Weighted models were used to estimate the association between surgical approach and both primary and secondary outcomes. CUSUM analysis of operative time was performed to evaluate the learning curve for each technique. CUSUM analysis was intended as an exploratory tool to describe procedural stabilization within each technique, rather than to provide a direct quantitative comparison between platforms and should not be interpreted as a definitive characterization of the learning curve. CUSUM plots display the cumulative deviation of each operative time from a predefined target value (here, the median operative time for each approach). An upward trend indicates longer-than-expected operative times, whereas a downward trend suggests improving efficiency. A plateau or inflection point is commonly interpreted as procedural stabilization.

For the analysis of NASA-TLX data, individual domain scores and the overall workload index (mean of all six domains) were calculated for each surgeon. Because of the small sample size (*n* = 4) and non-normal distribution, comparisons between the two robotic systems were performed using the Wilcoxon signed-rank test for paired data. Effect sizes were estimated using rank-biserial correlation coefficients (r). Results were graphically represented using radar charts, where higher scores indicated greater perceived workload. *p* values < 0.05 were considered statistically significant. Analyses were performed using SPSS (version 29) and JASP (version 0.19.1). Given the chronological separation between groups, a potential time-trend bias was considered. Although perioperative protocols remained stable, temporal improvements in institutional experience and perioperative care cannot be fully excluded and may have contributed to the observed differences.

### Ethical considerations

The study was conducted in accordance with the principles of the Declaration of Helsinki (2013 revision) and followed the Strengthening the Reporting of Observational Studies in Epidemiology (STROBE) guidelines. Approval was obtained from the Institutional Review Board/Ethics Committee of Milano Area 2 (protocol 662_2022, date: July 5, 2022). Written informed consent for surgery and for the use of anonymized clinical data for research purposes was obtained from all patients. Data confidentiality was guaranteed according to current European regulations (EU General Data Protection Regulation 2016/679).

## Results

**Figure 1:**
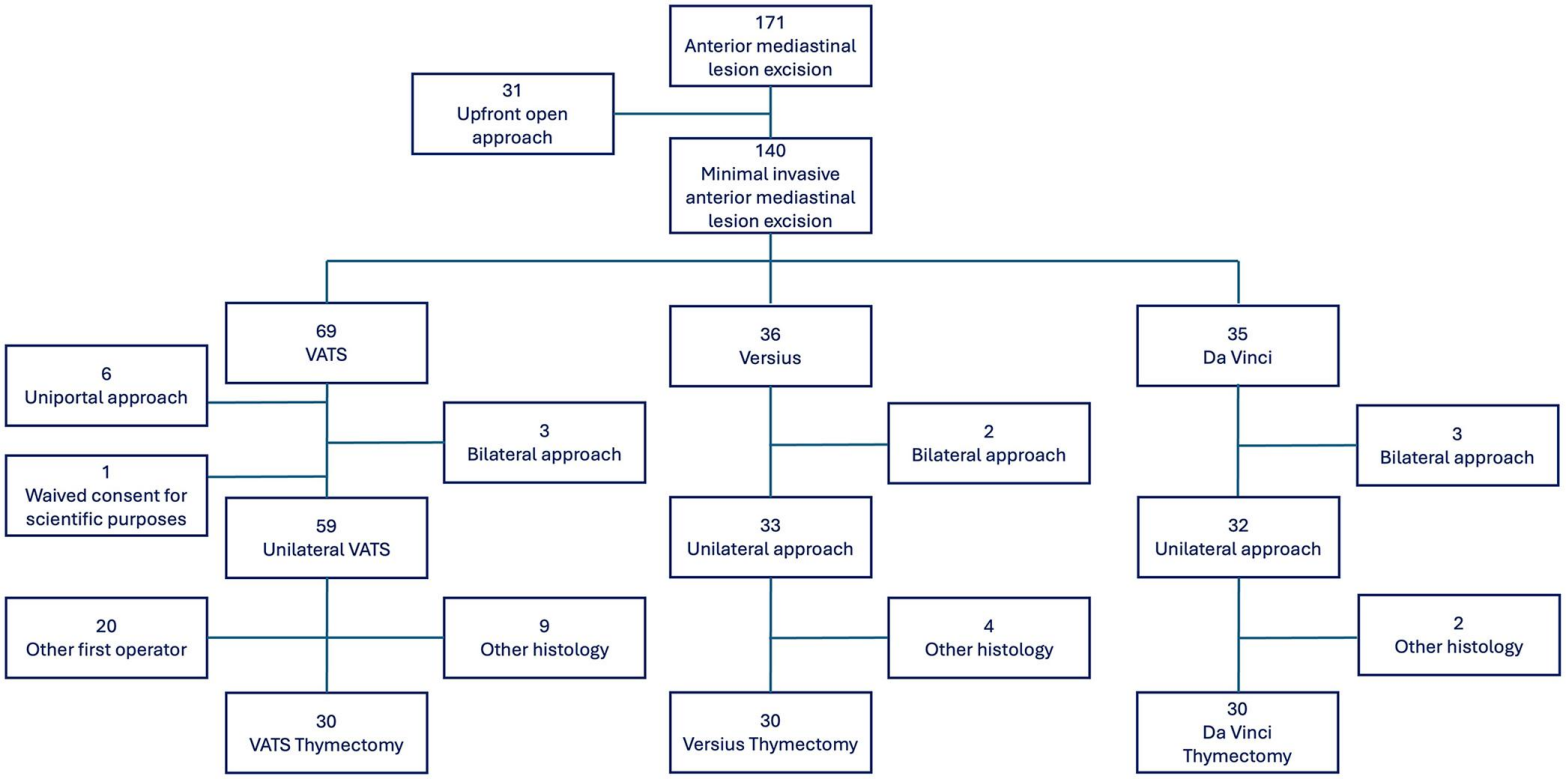
Flow chart illustrating patient selection. Among 171 patients who underwent thymectomy between 2016 and 2025, 90 met the inclusion criteria and were analyzed. Patients were allocated to three groups according to the surgical approach: video-assisted thoracoscopic surgery (VATS, *n* = 30), robot-assisted thoracoscopic surgery using the Da Vinci platform (RATS Da Vinci, *n* = 30), and robot-assisted thoracoscopic surgery using the Versius platform (RATS Versius, *n* = 30).

A total of 90 patients were included in the study (30 for each approach: VATS, RATS Da Vinci, and RATS Versius). Figure 1 illustrates the flow chart of patient selection. Baseline demographic characteristics were comparable across groups (Table 1). Mean age did not differ significantly between techniques (*p* = 0.565), and sex distribution was homogeneous (*χ*^2^ = 0.278, *p* = 0.870). Although ASA distribution did not reach statistical significance, a higher proportion of ASA class 3 patients was observed in the VATS group. Dimensions differed significantly across surgical approaches [F (2.32) = 4.89, *p* = 0.014]. In *post-hoc* pairwise comparisons with Šidák correction, VATS approach was associated with a significantly smaller dimension compared with Da Vinci approach (mean difference −2.11, 95% CI −3.90 to −0.31; *p* = 0.017). No significant differences were observed between Versius and Da Vinci approach or between Versius and VATS approach (Supplementary Table S1). Detailed intraoperative descriptions of pleural adhesions were not systematically recorded and could not be analyzed; stage IIIa thymoma included lung invasion in all the three reported cases.

**Table 1:** Patients' preoperative characteristics.

Variable	Da Vinci (*n* = 30)	VATS (*n* = 30)	Versius (*n* = 30)	*P* value
Age, years, mean (SD)	55.5 (15.79)	53.4 (13.07)	57.7 (17.41)	0.565
Sex				0.870
Male, *n* (%)	11.0 (36.7)	12 (40.0)	13 (43.3)	
Smoke				0.749
Yes, *n* (%)	4 (13.3)	3 (10.0)	7 (23.3)	
No, *n* (%)	19 (63.4)	20 (66.7)	17 (56.7)	
Former, *n* (%)	7 (23.3)	7 (23.3)	6 (20.0)	
Charlson Comorbidity Index, mean (SD)	2.9 (2.6)	1.6 (1.5)	2.5 (1.7)	0.532
Myasthenia Gravis, *n* (%)	10 (33.3)	14 (46.7)	10 (33.3)	0.863
ASA score				0.478
1, *n* (%)	0 (0.0)	0 (0.0)	0 (0.0)	
2, *n* (%)	17 (56.7)	6 (20.0)	21 (70.0)	
3, *n* (%)	12 (40.0)	24 (80.0)	9 (30.0)	
4, *n* (%)	1 (3.3)	0 (0.0)	0 (0.0)	
Diagnosis				0.921
Thymoma, *n* (%)	14 (46.7)	14 (46.7)	16 (53.3)	
Thymic Hyperplasia, *n* (%)	16 (53.3)	16 (53.3)	14 (46.7)	
Thymoma stage				0.836
I, *n* (%)	13 (92.9)	12 (85.7)	14 (87.5)	
II, *n* (%)	0 (0.0)	2 (14.3)	0 (0.0)	
IIIa, *n* (%)	1 (7.1)	0 (0.0)	2 (12.5)	
Thymoma resection status				0.907
R0, *n* (%)	13 (92.9)	13 (92.9)	15 (93.8)	
R1, *n* (%)	1 (7.1)	1 (7.1)	1 (6.2)	
Thymoma dimension, cm, mean (SD)	5.58 (2.40)	3.47 (0.76)	4.38 (2.16)	0.014

Table 2 summarizes the main outcomes. The primary endpoint, postoperative length of stay, showed significant differences among groups (ANOVA: *F* = 9.812, *p* < 0.001, *η*^2^ = 0.184), as shown in central picture. Patients undergoing VATS had a longer mean hospital stay (4.47 ± 1.85 days) compared with Da Vinci (3.20 ± 0.96 days, *p* = 0.002) and Versius (3.00 ± 1.20 days, *p* < 0.001). No significant differences were observed between the two robotic systems (*p* = 0.843). These findings were confirmed by non-parametric analysis (Kruskal–Wallis, *p* < 0.001). After propensity-weighted adjustment, the association between surgical approach and length of stay remained significant. In this regard, length of stay should be interpreted as a composite outcome reflecting surgical approach, team experience, and evolving institutional practices.

**Table 2:** Patients' perioperative outcomes.

Variable	Da Vinci *n* = 30	VATS *n* = 30	Versius *n* = 30	*P* value
Operative time, minutes, mean (SD)	183.5 (47.2)	165.4 (74.6)	182.7 (50.2)	0.406
Intraoperative complications, yes, *n* (%)	0.0 (0.0)	0.0 (0.0)	0.0 (0.0)	1.000
Conversion, yes, *n* (%)	0.0 (0.0)	2.0 (6.7)	2.0 (6.7)	0.394
Postoperative complications, yes, *n* (%)	3.0 (10.0)	2.0 (6.7)	2.0 (6.7)	0.856
Chest-tube removal, days, mean (SD)	1.7 (0.9)	2.1 (0.9)	1.8 (0.9)	0.261
Hospital length of stay, days, mean (SD)	3.2 (1.0)	4.5 (1.6)	3.0 (1.2)	<0.001
ME/day, value, mean (SD)	11.4 (4.4)	10.0 (4.8)	9.9 (3.6)	0.330

Regarding secondary outcomes, no significant differences were found among the three approaches in terms of operative time (165.4 ± 74.6 min for VATS, 183.5 ± 47.2 min for Da Vinci, 182.7 ± 50.2 min for Versius; *p* = 0.406), postoperative analgesic consumption (mean morphine-equivalents/day: 10.0 ± 4.8 for VATS, 11.4 ± 4.4 for Da Vinci, 9.9 ± 3.6 for Versius; *p* = 0.330), or chest tube removal time (2.10 ± 0.89 days for VATS, 1.73 ± 0.87 days for Da Vinci, 1.83 ± 0.91 days for Versius; *p* = 0.261). No significant differences in operative time were observed according to laterality.

When considering conversion to open surgery only (thoracotomy), rates were 6.7% for VATS (one vascular invasion, one technical problem of the camera), 0% for Da Vinci, and 3.3% for Versius (one pericardial invasion). In addition, one patient (3.3%) required conversion to VATS procedure due to technical problems of the Versius console. No conversions to sternotomy were observed.

Conversion to open surgery occurred in 6.7% of VATS cases, while conversion to VATS or open surgery was required in 6.7% of Versius cases. No conversions were recorded in the Da Vinci group (*χ*^2^ = 4.093, *p* = 0.394). Postoperative complications were rare overall and did not differ significantly between groups (*χ*^2^ = 0.310, *p* = 0.856).

**Figure 2:**
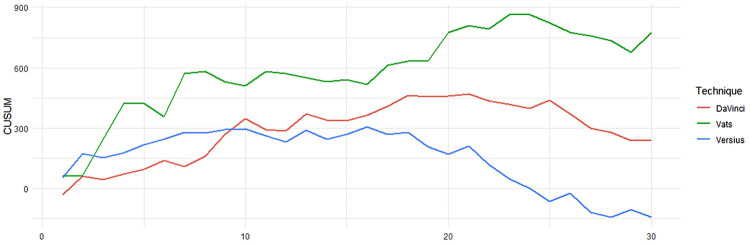
CUSUM curves. The cumulative sum (CUSUM) of operative time is plotted against the sequence of consecutive cases for each surgical technique. The target reference was set at the median operative time for each approach. The VATS curve (green) shows a progressive upward trend without reaching a plateau, indicating greater variability and a longer learning process. The Da Vinci curve (red) demonstrates an initial rise followed by stabilization after approximately 15 cases, suggesting achievement of procedural consistency. The Versius curve (blue) shows a rapid decline and early plateau formation, consistent with a shorter learning phase.

CUSUM analysis of operative times demonstrated different learning curve patterns among the three techniques (Figure 2). VATS showed a steady upward trend without reaching a plateau, suggesting greater variability and less evidence of procedural consolidation within the first 30 cases. Da Vinci displayed an initial increase followed by stabilization after approximately 15 cases, indicating progressive consolidation of operative performance. Versius showed earlier operative time stabilization in this specific setting, approaching a plateau around the 20th case.

**Figure 3:**
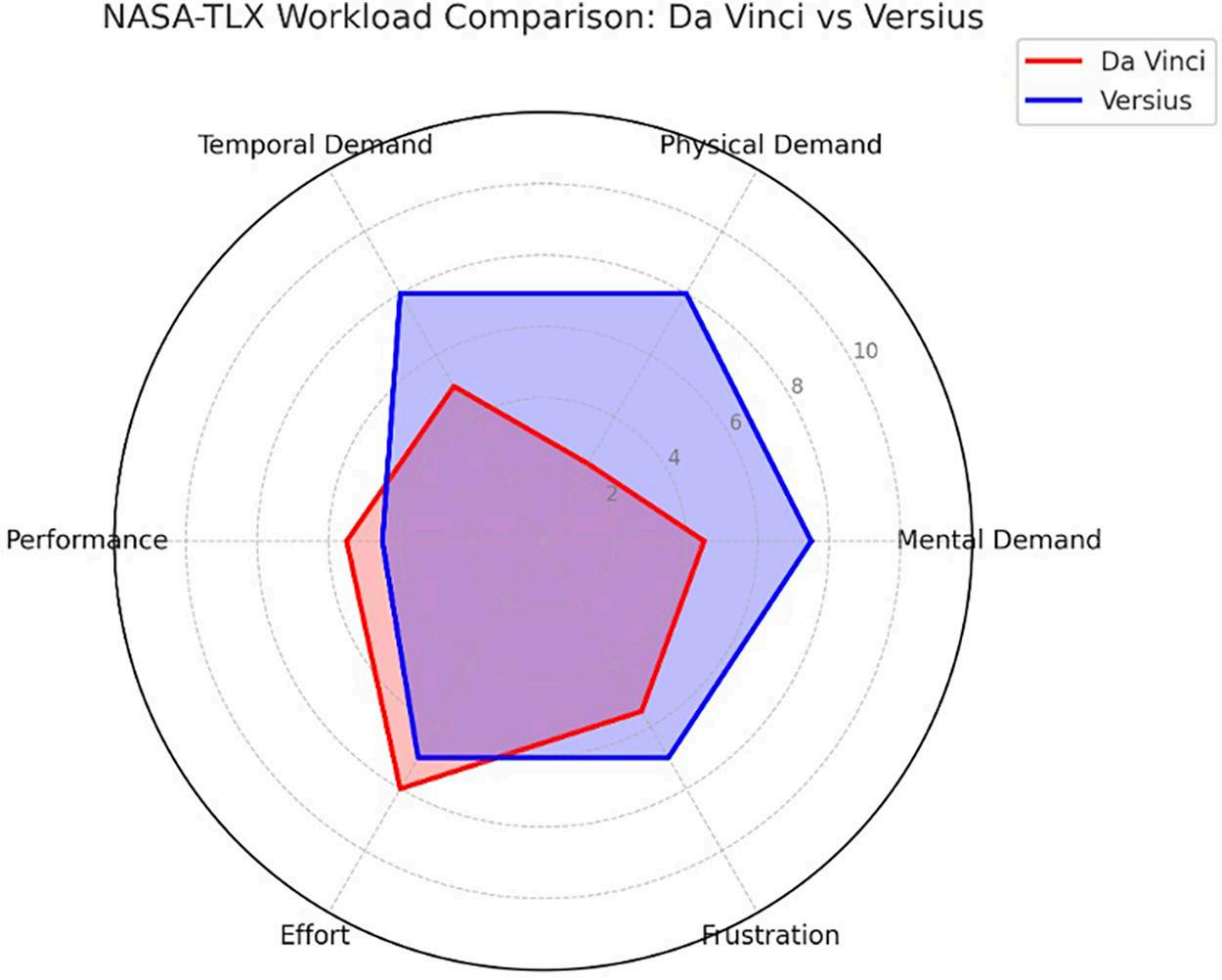
NASA Task Load Index (NASA-TLX) radar chart comparing perceived workload between the Da Vinci and versius robotic systems. Each axis represents one of the six workload dimensions: mental demand, physical demand, temporal demand, performance, effort, and frustration. Values range from 1 (lowest perceived workload) to 7 (highest perceived workload). The chart displays mean scores for each domain, illustrating higher overall workload perception with the Versius system.

Mean NASA-TLX scores indicated a higher overall workload perception with the Versius system (mean 7.0 ± 1.1) compared with the Da Vinci system (mean 5.2 ± 1.0). Versius surgeons reported greater mental and physical demands, whereas perceived performance was similar between platforms. These findings suggest a steeper early adaptation phase with the Versius system, consistent with the corresponding CUSUM learning curve patterns and the subjective workload profile illustrated in Figure 3.

## Comments

Although minimally invasive approaches have been extensively compared with open surgery in thymectomy (10–12), to the best of our knowledge, this is the first study to directly compare two different robotic platforms and VATS in thymic surgery, providing novel insights into both the clinical and ergonomic aspects of minimally invasive thymectomy.

The results confirm the safety and effectiveness of all three techniques, while highlighting shorter hospital stays and faster procedural consolidation with robotic systems compared with the VATS approach. However, these findings should be interpreted within a real-world, evolving institutional context, where surgical experience, team maturation, and temporal factors may interact with the intrinsic characteristics of each surgical approach.

The primary endpoint, postoperative length of stay, was significantly reduced in both robotic groups compared with VATS, with no difference between the two robotic platforms. These findings are consistent with previous reports demonstrating that robot-assisted thymectomy facilitates faster recovery and shorter hospitalization owing to superior visualization, precision, and tissue handling (5, 6). Notwithstanding, the observed reduction in length of stay in the robotic cohorts should be interpreted with caution, as part of this difference may reflect temporal improvements in perioperative management and institutional experience rather than the surgical platform alone. The higher proportion of ASA class 3 patients and patients with myasthenia gravis in the VATS group may have contributed to longer hospitalization, independent of surgical approach. The comparable results between the Da Vinci and Versius systems indicate that new-generation robotic platforms can achieve similar patient outcomes, despite differences in design and console ergonomics.

Although thymoma size differed among approaches, this did not translate into differences in operative time or complication rates. No significant differences were found among the three approaches in terms of operative time, postoperative analgesic requirements, complication rates, or conversion to open surgery. Conversions were mainly related to local invasion or technical issues rather than platform-specific limitations. These results support the notion that both VATS and RATS are safe and effective for the treatment of early-stage thymic disease, in agreement with prior meta-analyses (2, 13–16). The absence of differences in operative time between Da Vinci and Versius also suggests that the transition to a new robotic system does not entail a substantial time penalty once basic familiarity is achieved.

CUSUM analysis demonstrated distinct learning curve patterns among the three techniques. The VATS group did not reach a clear plateau within the first 30 cases, reflecting the broader heterogeneity of the thoracoscopic approach and its well-documented variability across surgeons (3, 4). In contrast, RATS Da Vinci showed a typical two-phase pattern, with stabilization of operative times after approximately 15 cases, consistent with previous single-surgeon studies reporting a relatively steep but short learning curve (6). Notably, the Versius platform exhibited a rapid decline in operative time and early plateau formation, suggesting a potentially faster adaptation phase. Differences in learning-curve patterns should be interpreted cautiously. A learning-curve bias is inherent to the study design, as surgeons had already accrued experience with thymectomy during the VATS phase before transitioning to robotic platforms. This prior experience, together with case chronology and team maturation, likely facilitated a smoother adaptation to robotic systems. Therefore, CUSUM findings describe procedural stabilization within each technique rather than intrinsic efficiency of one platform over another.

However, this interpretation must be balanced with the subjective data from the NASA-TLX analysis, which revealed higher perceived workload among Versius users, especially in terms of mental and physical demand highlighting the importance of integrating human-factor metrics into future comparative studies of surgical technologies. Together, these findings suggest that although Versius allows efficient task execution early in the learning process, the cognitive and physical adaptation required may initially be greater than with the Da Vinci system.

Assessment of surgeon workload using the NASA-TLX tool provided valuable insights into the human factors dimension of robotic thymectomy. Despite the higher perceived workload with Versius, self-rated performance scores were comparable between the two platforms. The combination of objective learning-curve data and subjective workload measures reinforces the importance of ergonomic and cognitive considerations in the evaluation of surgical technologies. These findings should be interpreted as exploratory given the very limited number of surgeons involved. Future studies with larger samples and longitudinal assessments are warranted to clarify how experience modulates workload perception over time.

This study has several limitations. Its retrospective design and the relatively small sample size may limit the generalizability of the findings. Although propensity score–based weighting was applied, residual confounding due to unmeasured variables cannot be excluded. The comparison between robotic platforms was influenced by the involvement of different surgical teams, even though perioperative management and institutional protocols were standardized. Therefore, differences between robotic platforms may partly reflect surgeon-specific factors rather than intrinsic technological differences. Additional intraoperative variables such as estimated blood loss were not consistently recorded. Consequently, the study was not powered to detect differences in low frequency but clinically relevant events such as conversion, postoperative myasthenic crisis, or reoperation. However, no intraoperative complications or conversions related to bleeding were observed. Additionally, surgeon workload assessment was limited to the early learning phase and to a small number of participants, warranting caution in the interpretation of these exploratory results. Surgeon workload was not assessed for the VATS approach, representing a missed opportunity to compare subjective workload across all techniques. Furthermore, the present analysis focused exclusively on short-term perioperative outcomes; oncologic and neurological follow-up data were not considered, as the study design was not intended to assess long-term results. Future prospective studies should include post-discharge analgesic consumption and patient-reported pain outcomes.

Moreover, this study focused exclusively on the lateral transthoracic approach, without including other minimally invasive access routes such as the subxiphoid or transcervical approaches, which may have distinct technical and ergonomic characteristics (17–25). In particular, the subxiphoid approach has gained increasing interest in minimally invasive thymic surgery and may offer advantages in selected settings, particularly regarding postoperative pain. However, current evidence does not support its universal superiority over lateral intercostal access for all perioperative outcomes, and approach selection remains strongly influenced by case characteristics and institutional expertise (26, 27).

Although a formal cost analysis was beyond the scope of this study, cost considerations are increasingly relevant when comparing robotic platforms. Previous reports suggest that newer-generation robotic systems may be associated with lower acquisition and maintenance costs compared with established platforms. In this context, the observation of comparable clinical outcomes between the Da Vinci and Versius systems may have important implications for the economic sustainability and broader adoption of robotic thymectomy. Nevertheless, these considerations should be interpreted cautiously given the limited sample size and the absence of a dedicated cost-effectiveness analysis.

In a real-world, evolving institutional setting, robotic thymectomy was associated with shorter hospital stay and earlier procedural stabilization compared with VATS, with comparable perioperative safety. Differences between robotic platforms were mainly related to surgeon-reported workload rather than clinical outcomes. These findings highlight the importance of considering surgeon experience, ergonomics, and institutional context when adopting new robotic technologies.

## Data Availability

The raw data supporting the conclusions of this article will be made available by the authors, without undue reservation.
