# Supplementary material for: Minimally invasive thymectomy: comparative analysis of short-term outcomes after video-assisted thoracoscopy and two robotic platforms
Source: Front Surg. 2026 May 25;13:1836452. doi: 10.3389/fsurg.2026.1836452 (PMC13243205; doi:10.3389/fsurg.2026.1836452)
Supplement: Supplementary file 1 [file Supplementaryfile1.docx]

Supplementary material

This supplementary section provides a detailed description of the standardized surgical techniques adopted at the Foundation IRCCS Ca’ Granda Ospedale Maggiore Policlinico for minimally invasive thymectomy. All procedures were performed by dedicated thoracic surgeons following consistent institutional protocols for patient positioning, anesthesia, and perioperative management.

*Surgical techniques.* All patients underwent thymectomy via a minimally invasive approach under general anesthesia with selective single-lung ventilation.

*VATS thymectomy.* VATS thymectomy was performed using a three-port approach, predominantly in the left hemithorax. Standard thoracoscopic instruments were used. The thymus and mediastinal fat were dissected from the pericardium, pleura, and great vessels and removed en bloc.

*RATS Da Vinci thymectomy.* Robot-assisted thymectomy with the Da Vinci Xi platform was performed using a three-arm configuration. The patient was positioned in a 30° lateral decubitus position, with the left arm placed alongside the body to allow unobstructed access to the left hemithorax. Three 8-mm robotic trocars were inserted under direct thoracoscopic vision along the left hemithorax as follows: the camera port was positioned in the 5th intercostal space at the anterior axillary line, the superior port in the 3rd intercostal space anterior to the camera port, and the inferior port in the 7th intercostal space. After entry into the pleural cavity, carbon dioxide insufflation was initiated at a pressure of 8–10 mmHg to enhance visualization and increase the working space within the anterior mediastinum. The Da Vinci Xi system was then docked from the patient’s right side.

*RATS Versius thymectomy*. Robot-assisted thymectomy with the Versius platform was performed using three bedside units positioned on the patient’s left side. The modular design allowed flexible port placement and bedside unit orientation. The patient’s position was the same as for the Da Vinci procedure, except that the left arm was raised above the head. Trocar placement and the main surgical steps mirrored those of the Da Vinci approach.

*Specimen retrieval.* In all groups, the specimen was placed in a protective bag and extracted by slightly enlarging the caudal thoracoscopic port as needed to allow safe removal.

No significant intraoperative modifications were made to the described techniques during the study period.

Table S1 Thymoma dimension according to approach.

| **Approach** | **Delta-method** | | **Sidak** | | **Sidak** |
| --- | --- | --- | --- | --- | --- |
|  | **Contrast** | **std. err.** | **t** | **P>\|t\|** | **[95% conf. interval]** |
| VATS vs Da Vinci | -2.106 | 0.711 | -2.96 | 0.017 | -3.899 -0.314 |
| Versius vs Da Vinci | -1.201 | 0.915 | -1.31 | 0.486 | -3.509 1.105 |
| Versius vs VATS | 0.905 | 0.668 | 1.35 | 0.459 | -0.779 2.589 |

Robust standard errors were used to account for heteroscedasticity.

Table S2. Baseline characteristics by surgical approach before and after IPTW, with standardized mean differences

| **Covariate** | **VATS Unweighted** | **Da Vinci Unweighted** | **Versius Unweighted** | **VATS Weighted** | **Da Vinci Weighted** | **Versius Weighted** | **SMD Unweighted** | **SMD Weighted** |
| --- | --- | --- | --- | --- | --- | --- | --- | --- |
| **Age, mean ± SD** | 53.40 ± 13.08 | 55.50 ± 15.79 | 57.70 ± 17.42 | 55.26 ± 13.14 | 54.25 ± 15.07 | 54.73 ± 19.87 | 0.28 | 0.07 |
| **BMI, mean ± SD** | 24.12 ± 3.23 | 25.65 ± 5.21 | 25.78 ± 4.21 | 24.45 ± 2.94 | 25.22 ± 4.88 | 25.84 ± 4.29 | 0.44 | 0.38 |
| **Charlson Comorbidity Index, mean ± SD** | 1.63 ± 1.47 | 2.90 ± 2.63 | 2.53 ± 1.72 | 1.87 ± 1.44 | 2.41 ± 2.31 | 2.16 ± 1.71 | 0.60 | 0.28 |
| **Male sex, %** | 40.00 | 36.70 | 43.30 | 31.40 | 41.70 | 47.30 | 0.13 | 0.33 |
| **Smoking (current/former), %** | 33.30 | 36.60 | 43.30 | 27.20 | 37.40 | 35.50 | 0.22 | 0.19 |
| **Myasthenia gravis, %** | 46.70 | 33.30 | 33.30 | 38.60 | 34.50 | 40.90 | 0.27 | 0.12 |
| **Thymoma diagnosis, %** | 53.30 | 53.30 | 46.70 | 50.70 | 56.00 | 49.50 | 0.00 | 0.09 |
| **ASA class ≥3, %** | 80.00 | 43.30 | 30.00 | 63.40 | 41.10 | 53.80 | 0.86 | 0.27 |

Weighted values were obtained using inverse probability of treatment weighting (IPTW). For the three-level exposure, the maximum absolute pairwise standardized mean difference (VATS vs Da Vinci, VATS vs Versius, Da Vinci vs Versius) is reported.

Figure S1


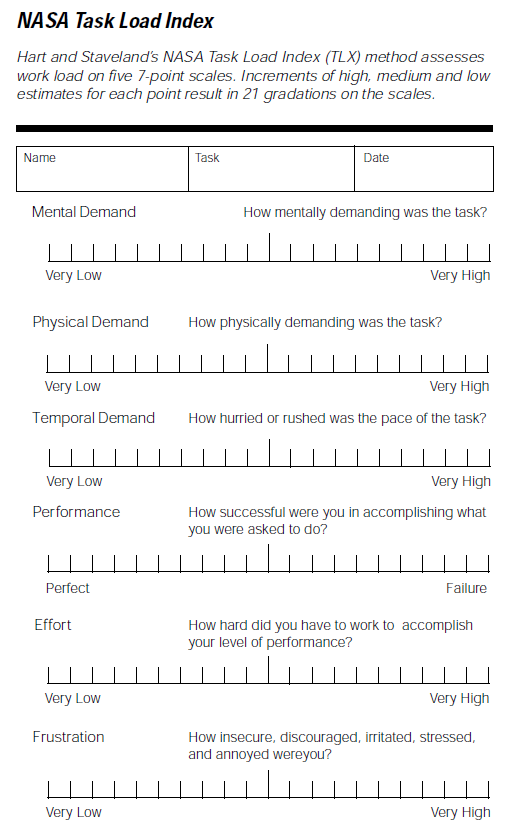


The NASA-TLX questionnaire is widely used in human factors research to assess perceived workload in complex tasks, including surgery. This validated, multidimensional tool measures subjective workload across six domains: mental demand, physical demand, temporal demand, performance, effort, and frustration. Each domain is rated on a 7-point scale, with higher scores indicating greater perceived workload.
